# Development of a Korean Fracture Risk Score (KFRS) for Predicting Osteoporotic Fracture Risk: Analysis of Data from the Korean National Health Insurance Service

**DOI:** 10.1371/journal.pone.0158918

**Published:** 2016-07-11

**Authors:** Ha Young Kim, Eun Jin Jang, ByeongJu Park, Tae-Young Kim, Soon-Ae Shin, Yong-Chan Ha, Sunmee Jang

**Affiliations:** 1 Department of Internal Medicine, Wonkwang University Sanbon Hospital, Gunpo, Republic of Korea; 2 Department of information Statistics, Andong National University, Andong, South Korea; 3 Department of Statistics, Kyungpook National University, Daegu, South Korea; 4 Department of Orthopaedic Surgery, School of Medicine, Hallym University, Anyang, Korea; 5 Big Data Steering Department, National Health Insurance Service, Seoul, Korea; 6 Department of Orthopaedic Surgery, Chung-Ang University College of Medicine, Seoul, South Korea; 7 College of Pharmacy, Gachon University, Incheon, Korea; Garvan Institute of Medical Research, AUSTRALIA

## Abstract

**Background:**

Asian-specific prediction models for estimating individual risk of osteoporotic fractures are rare. We developed a Korean fracture risk prediction model using clinical risk factors and assessed validity of the final model.

**Methods:**

A total of 718,306 Korean men and women aged 50–90 years were followed for 7 years in a national system-based cohort study. In total, 50% of the subjects were assigned randomly to the development dataset and 50% were assigned to the validation dataset. Clinical risk factors for osteoporotic fracture were assessed at the biennial health check. Data on osteoporotic fractures during the follow-up period were identified by ICD-10 codes and the nationwide database of the National Health Insurance Service (NHIS).

**Results:**

During the follow-up period, 19,840 osteoporotic fractures were reported (4,889 in men and 14,951 in women) in the development dataset. The assessment tool called the Korean Fracture Risk Score (KFRS) is comprised of a set of nine variables, including age, body mass index, recent fragility fracture, current smoking, high alcohol intake, lack of regular exercise, recent use of oral glucocorticoid, rheumatoid arthritis, and other causes of secondary osteoporosis. The KFRS predicted osteoporotic fractures over the 7 years. This score was validated using an independent dataset. A close relationship with overall fracture rate was observed when we compared the mean predicted scores after applying the KFRS with the observed risks after 7 years within each 10th of predicted risk.

**Conclusion:**

We developed a Korean specific prediction model for osteoporotic fractures. The KFRS was able to predict risk of fracture in the primary population without bone mineral density testing and is therefore suitable for use in both clinical setting and self-assessment. The website is available at http://www.nhis.or.kr.

## Introduction

Osteoporosis is characterized by low bone mass, microarchitectural deterioration of bone tissue, and reduced bone quality [1]. The importance of this disease arises from its complication of fragility fractures which are associated with high morbidity and mortality. Osteoporotic fractures have become a major health and economic burden in Asian countries as in North America and Europe. With the aging population rapidly increasing in Asia, it is projected that by 2050, half of the world’s hip fractures will occur in Asians[2]. In Korea, 12.3% of women aged 50 years experiences a hip fracture in their life. In addition, 59.5% have osteoporotic fractures during their lifetime[3]. The socioeconomic burden of osteoporotic fractures is predicted to increase dramatically in the future because the rate of increase in the elderly population in Korea is greater than that of elsewhere. Therefore, early detection of individuals with high fracture risk would have a substantial impact on reducing the burden caused by fractures in Korea.

Low bone mineral density (BMD) is a strong predictor of osteoporotic fracture risk [4]. However, BMD alone is insufficient to identify all individuals with high risk because osteoporotic fractures can occur in patients with any given T-score [5], and even in those with normal BMD values, according to the current World Health Organization (WHO) classification. Thus, a number of clinical risk factors that provide information on fracture risk independent of BMD have been identified [6–13]. Recently, several algorithms have been developed to estimate fracture probability using additional risk factors for fracture. Among these algorithms, the WHO Fracture Risk Assessment Tool (FRAX) algorithm[14], Q fracture algorithm[15], and Garvan Fracture Risk Calculator(Garvan)[16, 17] are widely available and used. Several studies have compared various tools for their ability to identify women at highest risk of fracture[18–20]. Most of these studies reached the conclusions that the simpler tools perform as well as the more complex tools. The FRAX algorithm, which has been incorporated into several national guidelines, provides 10-year absolute fracture risk utilizing a set of clinical risk factors with or without BMD data in different populations[14], including Korea. These factors include low body mass index (BMI), current smoking, mean alcohol intake of three or more units daily, parental history of hip fracture, prior fragility fracture, long-term use of oral glucocorticoids, rheumatoid arthritis, and other secondary causes of osteoporosis. However, the clinical risk factors included in FRAX are slightly different than those identified in prospective population studies [15, 16, 21, 22]. The risk and incidence of osteoporotic fractures varies widely between populations [23]. Thus, ethnic- and other population-specific data are needed to effectively predict new fracture risk in a given population. However, few studies have investigating the clinical risk factors of osteoporotic fractures in Korea.

The main concern when managing osteoporosis is identifying individuals at high risk for pharmacological intervention. To do this, better risk assessment tools are needed to enhance fracture predictions. A nationwide database is required to develop a fracture risk assessment tool for Korea. The Korean government operates a mandatory national health insurance system with a central database called the Nation Health Insurance Service (NHIS). This database contains all prescription drug and treatment claim records for almost all Koreans. The purpose of our study was to develop and validate a practical tool for osteoporotic fracture risk assessment using this Korean nationwide database.

## Materials and Methods

### Study Population

The Korean NHIS provides health insurance to all Koreans, and all Koreans are obliged to become members of this national insurance system. The Korean NHIS performs the National Health Checkup every 2 years to insured Koreans over 40 years, and this checkup is enforced by law. Local hospitals that are fully qualified based on the NHIS criteria perform these health checkups. The National Health Checkup includes height, weight, blood pressure measurements, chest radiography, urinalysis, blood count, and blood chemistry. In addition, a self-administered questionnaire is used to collect medical history, current health status, family history, tobacco and alcohol consumption, dietary preferences, and leisure-time physical activities.

Two million men and women were randomly selected from participants who had taken health checkups from January 1 to December 31, 2006. We excluded subjects aged < 50 and > 90 years (n = 1,269,844). In addition, subjects who had missing information for height or weight (n = 300) were excluded. There was no missing data in other variables. Subjects who had been prescribed osteoporosis medication (e.g., bisphosphonate, selective estrogen receptor modulator, calcitonin, or calcium /vitamin D) or who had used any of these medications > 30 days during the year prior to the health checkup (n = 11,550) were also excluded. The final study population consisted of 718,306 participants (370,242 men and 348,604 women) who were followed-up for 7 years. We linked all 718,306 patients from the National Health Checkup database to the KNHIS database to track the fracture occurrence. The Institutional Review Board of Chung-Ang University Hospital reviewed and approved this study (IRB No. C201486 (1282)).

### Clinical Risk Factor Assessment

Information on potentially significant clinical risk factors selected based on a reported association with osteoporotic fractures was obtained from self-administered questionnaires. The questionnaire included questions about age, smoking status, alcohol consumption, and exercise (frequency and duration). The participants were classified as “current smokers” if they were currently smoking for at least 1 year, “nonsmokers” if they had never smoked, and “former smokers: if they had quit smoking. Total daily alcohol intake was measured by the number of glasses of “Soju” consumed per week. One glass of Soju contains about one unit of ethanol. The recommended amount suggested by the WHO is < 5 units per day for men and 2.5 units per day for women. In this study, five or more units for men and three or more units for women were considered high alcohol intake. Exercise was categorized as none, 1–2 times per week, 3–4 times per week and daily.

Height and body weight were measured with the subjects wearing light clothes. BMI was calculated as weight divided by height squared (kg/m^2^), and weight was stratified into the following four categories; underweight, < 18.5 kg/m^2^; normal, 18.5–22.9 kg/m^2^; overweight, 23.0–24.9 kg/m^2^; and obese, ≥ 25.0 kg/m^2^ [24].

A history of recent fragility fracture, previous medication use, and other diseases that could cause osteoporosis were also investigated in the KNHIS database. The KNHIS data covers the entire population, including 97% of the population using health insurance and 3% who use medical aid[25]. All clinics and hospitals submit claims data for inpatient and outpatient care, including diagnoses (using the International Classification of Diseases, 10th revision [ICD-10] codes), procedures, prescription records, demographic information, and direct medical costs. Therefore, virtually all information about patients and their medical records are available. We analyzed the data from 2004 to 2006. History of a recent fragility fracture was defined if the fragility fracture (vertebrae, hip, upper arm, and wrist fracture) had occurred during the past two years before baseline. Recent use of an oral glucocorticoid was defined if it was prescribed for more than 30 days in the past year before baseline. The diagnosis of rheumatoid arthritis was defined if the subject visited the outpatient clinic more than twice or was admitted more than once using a related code in the 1 year before baseline. Pharmaceutical or medical conditions that cause secondary osteoporosis were also investigated. Medications included anticonvulsants, anticoagulants (e g, warfarin and heparin), aromatase inhibitors, and a suppressive dose of thyroid hormone. Medical conditions included thyrotoxicosis, hyperparathyroidism, hyperprolactinemia, hypopituitarism, Cushing’s syndrome, hypogonadism, r premature menopause (<45 years), chronic renal failure, chronic obstructive lung disease, bypass surgery, inflammatory bowel disease, multiple myeloma, and idiopathic hypercalciuria.

### Outcomes

Our primary outcome was the first diagnosis of an osteoporotic fracture (hip, vertebral, upper arm, or wrist)[26]. We identified all claims records of outpatient visits or hospital admissions of patients from January 1, 2006 to December 31, 2013 in the KNHIS data. We used particular ICD-10 codes and procedures to identify osteoporosis-related fractures. [27, 28]. These were hip (ICD-10 code S72.0 [fracture of the femoral neck], S72.1 [pertrochanteric fracture] and seven procedures [open reduction of fractured extremity-femur, closed pinning-femur, external fixation-pelvis/femur, closed reduction of fractured extremity-pelvis/femur, bone traction, skin traction, hemiarthroplasty-hip]); spine (S22.0 [fracture of the thoracic spine], S22.1 [multiple fractures of the thoracic spine], S32.0 [fracture of the lumbar spine], M48.4 [fatigue fracture of vertebra] and M48.5 [collapsed vertebra, NEC]); distal radius (S52.5 [fracture of the distal radius] and S52.6 [combined fracture of the distal radius/ulna]); humerus (S42.2 [fracture of the proximal humerus] and S42.3 [fracture of shaft of humerus]); and overall any fractures[3]. Each fracture code had to be accompanied by a physician’s claim for site-specific fracture reduction or fixation (either open or closed) to enhance the specificity of the coding. The total number of men and women > 50 years in the Korean population was obtained from Statistics Korea (http://www.kosis.kr/), which is the central governmental statistical organization.

### Model Development and Validation

The osteoporotic fracture predictive models for men and women were developed separately. We randomly divided the total male and female cohorts into a 50% modeling cohort and 50% validation cohort. Baseline characteristics are summarized. The mean and standard deviation were used for continuous variables while frequencies and percentages were used for categorical variables. The incidence rates and 95% confidence intervals (CIs) for spine fracture, hip fracture, and other fractures per 1,000 person-years were calculated according to age category. The following variables were initially identified from the literature as traditional risk factors for osteoporotic fracture: age, height, weight, prior fracture, current smoking status, use of steroids, rheumatoid arthritis, high alcohol intake, and exercise status. The predictive models were estimated using the Fine and Gray model by considering death as a competing risk in the modeling set. The risk might be overestimated in the predictive model using standard Cox’s proportional hazards regression model by treating death as a censored case because subjects with death events are considered as if they could have fracture in the future[29]. Therefore, the Fine and Gray model is used as the appropriate predictive model with competing risk. We followed each patient from the examination date in 2006 until the fracture or December 31, 2013. Patients who did not develop a fracture until the last day were censored. We considered pre-specified risk factors and all interaction terms in the predictive models.

Cumulative incidence function in the Fine and Gray model with k risk factors for time t (t = 7) was estimated for each sex using the following equation:

I(t|x)=1−exp(−exp[f(x,M)]S(t))

where *f*(*x*,*M*) = *β*_1_(*x*_1_−*M*_1_)+*β*_2_(*x*_2_−*M*_2_)+⋯+*β*_*k*_(*x*_*k*_−*M*_*k*_). Here, *β*_1_, *β*_2_, ⋯, *β*_*k*_ are regression coefficients; *x*_1_, *x*_2_, ⋯, *x*_*k*_ are risk factors for each individual; *M*_1_, *M*_2_, ⋯, *M*_*k*_ are mean values for each risk factor in the total cohort; and *S*(*t*) is the cumulative subdistribution baseline hazard at time t (t = 7). The mean follow-up period was 6.9 years in men and 6.6 years in women.

The proportionality assumptions in the Fine and Gray model for each variable were checked using Schoenfeld residuals plots. The subdistribution hazard ratios (sHRs) of the predictive model, 95% CIs, and *p*-values were presented.

The predictive accuracy for the occurrence of a fracture within a 7-year period was assessed for calibration and discrimination. We tested the performances of the final models using the validation dataset. Calibration or how closely the prediction reflected an actual event was assessed using the ratio of observed and predicted probabilities. We calculated observed probabilities using the cumulative incidence function estimate and the ratio of observed and predicted probabilities in deciles. Discrimination or the ability to distinguish between those who experienced the event and those who did not was assessed using c-statistics for the survival model with competing risk[29, 30]

All statistical analyses were performed using SAS ver. 9.4 (SAS Institute, Cary, NC, USA) and R package ver. 3.2.2. *P*-values < 0.05 were considered significant.

## Results

### Baseline Characteristics of the Development and Validation Groups

Overall, 718,306 subjects met the inclusion criteria, of which 50% were randomly assigned to the development dataset and the rest 50% were assigned to the validation dataset. The baseline characteristics of the two datasets are compared in Table 1. Mean age and BMI were similar between the two sexes. Clinical risk factors, such as current smoking, high alcohol intake, and one or more times of weekly exercise, were more common in men than those in women. However, risk factors, such as a recent fragility fracture, recent use of an oral glucocorticoid, rheumatoid arthritis, and other causes of secondary osteoporosis, were more common in women than those in men. Although the validation cohort was randomized as an independent group, the baseline characteristics were similar to those of the development cohort across all measures in men and women.

**Table 1:** Baseline characteristics of the study population.

Characteristics	Total cohort				Development cohort				Validation cohort			
	Men		Women		Men		Women		Men		Women	
	(N = 370,255)		(N = 348,253)		(N = 185,127)		(N = 174,126)		(N = 185,128)		(N = 174,127)	
	n	(%)	n	(%)	n	(%)	n	(%)	n	(%)	n	(%)
Age(year), Mean±SD	59.77±7.86		60.63±8.25		59.78±7.87		60.62±8.25		59.77±7.85		60.63±8.24	
50–59	204,508	(55.23%)	175,379	(50.36%)	102,363	(55.29%)	87,557	(50.28%)	102,145	(55.18%)	87,822	(50.44%)
60–69	114,832	(31.01%)	113,762	(32.67%)	57,169	(30.88%)	57,041	(32.76%)	57,663	(31.15%)	56,721	(32.57%)
70–79	44,827	(12.11%)	51,849	(14.89%)	22,517	(12.16%)	25,886	(14.87%)	22,310	(12.05%)	25,963	(14.91%)
80–89	6,088	(1.64%)	7,263	(2.09%)	3,078	(1.66%)	3,642	(2.09%)	3,010	(1.63%)	3,621	(2.08%)
BMI, Mean±SD	23.95±2.83		24.25±3.11		23.95±2.83		24.25±3.10		23.96±2.82		24.26±3.12	
< 18.5	9,171	(2.48%)	7,201	(2.07%)	4,563	(2.46%)	3,578	(2.05%)	4,608	(2.49%)	3,623	(2.08%)
18.5–22.9	123,322	(33.31%)	114,670	(32.93%)	61,870	(33.42%)	57,456	(33.00%)	61,452	(33.19%)	57,214	(32.86%)
23–24.9	106,285	(28.71%)	92,762	(26.64%)	53,056	(28.66%)	46,432	(26.67%)	53,229	(28.75%)	46,330	(26.61%)
≥ 25	131,477	(35.51%)	133,620	(38.37%)	65,638	(35.46%)	66,660	(38.28%)	65,839	(35.56%)	66,960	(38.45%)
Recent fragility fracture	1,815	(0.49%)	5,658	(1.62%)	887	(0.48%)	2,836	(1.63%)	928	(0.50%)	2,822	(1.62%)
Current smoking	143,928	(38.87%)	9,794	(2.81%)	71,884	(38.83%)	4,952	(2.84%)	72,044	(38.92%)	4,842	(2.78%)
High alcohol intake*	49,754	(13.44%)	5,770	(1.66%)	24,862	(13.43%)	2,874	(1.65%)	24,892	(13.45%)	2,896	(1.66%)
Weekly exercise of one or more times	188,769	(50.98%)	130,614	(37.51%)	94,530	(51.06%)	65,164	(37.42%)	94,239	(50.90%)	65,450	(37.59%)
Recent use of oral glucocorticoids	2,920	(0.79%)	3,541	(1.02%)	1,489	(0.80%)	1,780	(1.02%)	1,431	(0.77%)	1,761	(1.01%)
Rheumatoid arthritis	4,332	(1.17%)	11,429	(3.28%)	2,252	(1.22%)	5,629	(3.23%)	2,080	(1.12%)	5,800	(3.33%)
Other causes of secondary osteoporosis	78,961	(21.33%)	84,843	(24.36%)	39,520	(21.35%)	42,505	(24.41%)	39,441	(21.30%)	42,338	(24.31%)

* Five or more units for men, three or more units for women

### Fracture Incidence

The incidence rates of osteoporotic fracture in each cohort are shown in Table 2. During the follow-up period, 19,840 new osteoporotic fractures (4,889 in men and 14,951 in women) were reported in the development cohort. Fracture incidences per 1000 person-years were 12.09 (95% CI, 11.89–12.28) in women and 3.61 (95% CI, 3.51–3.71) in men. A total of 6,892 (46.1%) vertebral fractures, 1,403 (9.4%) hip fractures, and 7,555 (50.5%) humerus and wrist fractures occurred in women. In men, there were 2,481 (50.7%) vertebral fractures, 865 (19.3%) hip fractures, and 1,776 (39.6%) humerus and wrist fractures.

**Table 2:** Incidence of osteoporotic facture.

	Modeling cohort								Validation cohort							
	Osteoporotic		Spine		Hip		Others *		Osteoporotic		Spine		Hip		Others ^a^	
	No	Rate/1,000PY (95% CI)	No	Rate/1,000PY (95% CI)	No	Rate/1,000PY (95% CI)	No	Rate/1,000PY (95% CI)	No	Rate/1,000pPY (95% CI)	No	Rate/1,000PY (95% CI)	No	Rate/1,000PY (95% CI)	No	Rate/1,000PY (95% CI)
**Women**																
Total	14,951	12.09(11.89, 12.28)	6,892	5.42 (5.30, 5.55)	1,403	1.09 (1.03, 1.14)	7,555	5.96 (5.83, 6.10)	14,879	12.03 (11.84, 12.22)	6,925	5.45 (5.32, 5.58)	1,406	1.09 (1.03, 1.14)	7,554	5.96 (5.83, 6.10)
50–59	4,111	6.50 (6.30, 6.69)	1,069	1.66(1.56, 1.76)	118	0.18 (0.15, 0.21)	3,029	4.75 (4.59, 4.92)	4,175	6.58 (6.38, 6.78)	1,087	1.68 (1.58, 1.78)	121	0.19 (0.15, 0.22)	3,094	4.84 (4.67, 5.01)
60–69	5,699	14.12 (13.75, 14.49)	2,709	6.51 (6.26, 6.75)	392	0.92 (0.83, 1.01)	2,903	7.00 (6.75, 7.25)	5,600	13.95 (13.58, 14.32)	2,724	6.58 (6.34, 6.83)	385	0.91 (0.82, 1.00)	2,867	6.95 (6.70, 7.21)
70–79	4,341	24.59 (23.86, 25.32)	2,652	14.43 (13.88, 14.98)	659	3.43 (3.17, 3.69)	1,425	7.55 (7.16, 7.94)	4,314	24.33 (23.61, 25.06)	2,653	14.38 (13.83, 14.93)	683	3.54 (3.28, 3.81)	1,388	7.33 (6.94, 7.72)
80–89	800	33.47 (31.15, 35.79)	462	18.20 (16.54, 19.86)	234	8.84 (7.71, 9.97)	198	7.46 (6.43, 8.50)	790	33.42 (31.09, 35.75)	461	18.29 (16.62, 19.96)	217	8.24 (7.15, 9.34)	205	7.79 (6.72, 8.85)
**Men**																
Total	4,889	3.61 (3.51, 3.71)	2,481	1.82 (1.75, 1.89)	865	0.63 (0.59, 0.67)	1,776	1.30 (1.24, 1.36)	5,036	3.72 (3.62, 3.82)	2,609	1.91 (1.84, 1.99)	840	0.61 (0.57, 0.65)	1,800	1.32 (1.26, 1.38)
50–59	1,460	1.94 (1.85, 2.04)	582	0.77 (0.71, 0.83)	142	0.19 (0.16, 0.22)	777	1.03 (0.96, 1.10)	1,518	2.03 (1.93, 2.13)	583	0.77 (0.71, 0.84)	150	0.20 (0.17, 0.23)	819	1.09 (1.02, 1.16)
60–69	1,722	4.11 (3.92, 4.31)	891	2.11 (1.97, 2.25)	255	0.60 (0.53, 0.67)	640	1.51 (1.40, 1.63)	1,808	4.28 (4.09, 4.48)	958	2.25 (2.11, 2.39)	278	0.65 (0.57, 0.73)	635	1.49 (1.37, 1.60)
70–79	1,415	8.67 (8.22, 9.12)	832	5.02 (4.68, 5.36)	383	2.29 (2.06, 2.51)	314	1.87 (1.67, 2.08)	1,411	8.74 (8.28, 9.19)	883	5.39 (5.04, 5.75)	325	1.96 (1.74, 2.17)	292	1.76 (1.56, 1.96)
80–89	292	13.34 (11.81, 14.87)	176	7.85 (6.69, 9.01)	85	3.72 (2.93, 4.51)	45	1.96 (1.39, 2.53)	299	13.98 (12.40, 15.57)	185	8.46 (7.24, 9.68)	87	3.90 (3.08, 4.72)	54	2.41 (1.77, 3.05)

* Humerus and wrist fracture

Incidence rates were higher in women than in men and rose steeply with age. In the development cohort, fracture incidence rates per 1000 person-years according to age were 1.94, 4.11, 8.67, and 13.34 for men aged 50–59, 60–69, 70–79, and 80–89 years, respectively. The corresponding fracture incidences in women were 6.5, 14.12, 24.59, and 33.47, respectively. Similar incidence rates were found in the validation cohort (Table 2).

### Model Development

Results of the final Fine and Gray model for osteoporotic fractures in men and women are shown in Table 3. The interactions among all considered risk factors were tested. High daily alcohol intake and other causes of secondary osteoporosis were significant in the osteoporotic fracture prediction model for men. However, the coefficients and accuracy of the model without the interaction terms were similar to those of the model with interactions. Thus, the predictive models without the interactions were selected as the final models. There was no evidence that the proportionality assumption was not satisfied in any of these models.

**Table 3:** Hazard ratios (HR) for fracture*risk factors in men and women in the modeling cohort.

	Men			Women		
	HR	95% CI	*p*-value	HR	95% CI	*p*-value
Age						
50–59	1.00 (reference)			1.00 (reference)		
60–69	2.02	(1.88, 2.17)	< .0001	2.15	(2.06, 2.24)	< .0001
70–79	3.94	(3.65, 4.25)	< .0001	3.61	(3.45, 3.77)	< .0001
80–89	5.66	(4.96, 6.46)	< .0001	4.72	(4.37, 5.1)	< .0001
BMI						
< 18.5	1.65	(1.46, 1.86)	< .0001	1.12	(1.02, 1.24)	0.0180
18.5–22.9	1.00 (reference)			1.00 (reference)		
23–24.9	0.82	(0.76, 0.88)	< .0001	0.95	(0.91, 0.99)	0.0089
≥ 25	0.79	(0.73, 0.84)	< .0001	0.89	(0.86, 0.93)	< .0001
Recent fragility fracture	3.53	(2.91, 4.28)	< .0001	1.83	(1.67, 1.99)	< .0001
Current smoking	1.08	(1.02, 1.15)	0.0110	1.15	(1.05, 1.25)	0.0020
High alcohol intake**	1.37	(1.27, 1.48)	< .0001	1.20	(1.06,1.36))	0.0043
Weekly exercise of one or more times	0.76	(0.72, 0.81)	< .0001	0.87	(0.84, 0.9)	< .0001
Recent use of oral glucocorticoids	1.87	(1.53, 2.28)	< .0001	1.51	(1.33, 1.71)	< .0001
Rheumatoid arthritis	1.29	(1.06, 1.57)	0.0120	1.06	(0.97, 1.16)	0.2000
Other causes of secondary osteoporosis	1.10	(1.03, 1.17)	0.0069	1.07	(1.03, 1.11)	0.0010
c-index	0.68			0.65		

BMI = body mass index

* Fracture include spine, hip, humerus and wrist fractures

** Five or more units for men, three or more units for women

We found significant associations between overall fracture risk and age, BMI, recent fragility fracture, current smoking, high alcohol intake, exercise, recent use of oral glucocorticoids and other causes of secondary osteoporosis in both men and women after adjusting for all other variables in the models.

Age was the strongest predictor of any new fracture. Advanced age showed a trend of increased fracture risk. With the exception of age, recent fragility fracture and recent use of glucocorticoids were associated with the highest predicted 7-year risk of osteoporotic fracture: the hazard ratio (HR) was 3.53 and 1.87 in men 1.83 and 1.51 in women, respectively. Other risk factors listed in decreasing order of impact on fracture risk were BMI < 18.5 kg/m^2^, high alcohol intake, rheumatoid arthritis, secondary osteoporosis, and current smoking. Overweight, obesity and exercise at least once per week were protective factors for osteoporotic fracture risk, with HR of 0.82, 0.78 and 0.76 in men and 0.95, 0.89 and 0.87 in women, respectively. Rheumatoid arthritis was significantly associated with risk of osteoporotic fracture in men but not in women. The magnitude and direction of the coefficient was similar to those for overall risk of fracture, so it was included in the final osteoporotic fracture models for consistency.

**Fig 1:**
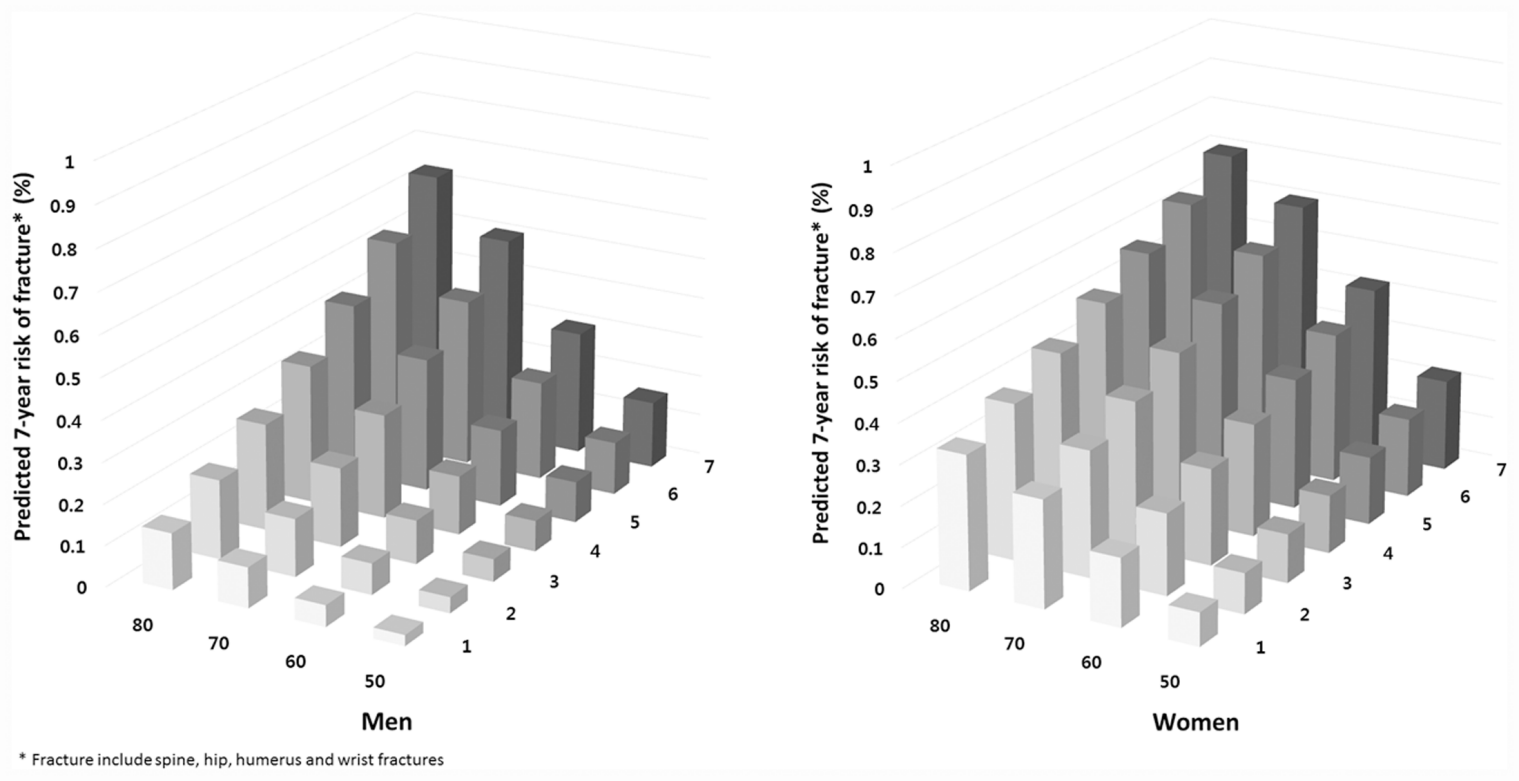
7- year osteoporotic fracture risk for Koreans according to age and number of risk fractors.

The impact of age on the risk of osteoporotic fracture was analyzed. Table 4 shows the 7- year fracture probability of various risk factors and how they were affected by age and sex. BMI is held constant in the normal range. As expected, the fracture risk increased with age in the absence of any clinical risk factors. The presence of any single risk factor increased fracture risk. The contributions of recent fragility fracture and recent use of oral glucocorticoid were attenuated with advancing age and were higher in men than in women. Fig 1 shows that the risk of major osteoporotic fractures was increased with increasing number of risk factors and age in men and women. Fracture probability at an advanced age was increased markedly with more risk factors.

**Table 4:** 7- year osteoporotic fracture risk * with normal BMI according to age and the absence or presence of single clinical risk factors.

	50	60	70	80
Men				
None	2.14	4.27	8.16	11.51
Recent fragility fracture	7.35	14.29	25.98	35.07
Current smoking	2.31	4.61	8.8	12.38
High alcohol intake*	2.93	5.82	11.04	15.46
Weekly exercise of one or more times	1.63	3.27	6.28	8.89
Recent use of oral glucocorticoids	3.95	7.82	14.69	20.4
Rheumatoid arthritis	2.75	5.47	10.39	14.57
Other causes of secondary osteoporosis	2.34	4.68	8.92	12.56
Women				
None	7.49	15.41	24.48	30.75
Recent fragility fracture	13.24	26.32	40.1	48.87
Current smoking	8.5	17.39	27.42	34.26
High alcohol intake*	8.9	18.17	28.57	35.62
Weekly exercise of one or more times	6.51	13.49	21.58	27.25
Recent use of oral glucocorticoids	11.08	22.32	34.54	42.57
Rheumatoid arthritis	7.91	16.25	25.73	32.25
Other causes of secondary osteoporosis	7.97	16.35	25.89	32.44

* Fracture include spine, hip, humerus and wrist fractures

### Validation of the KFRS

**Fig 2:**
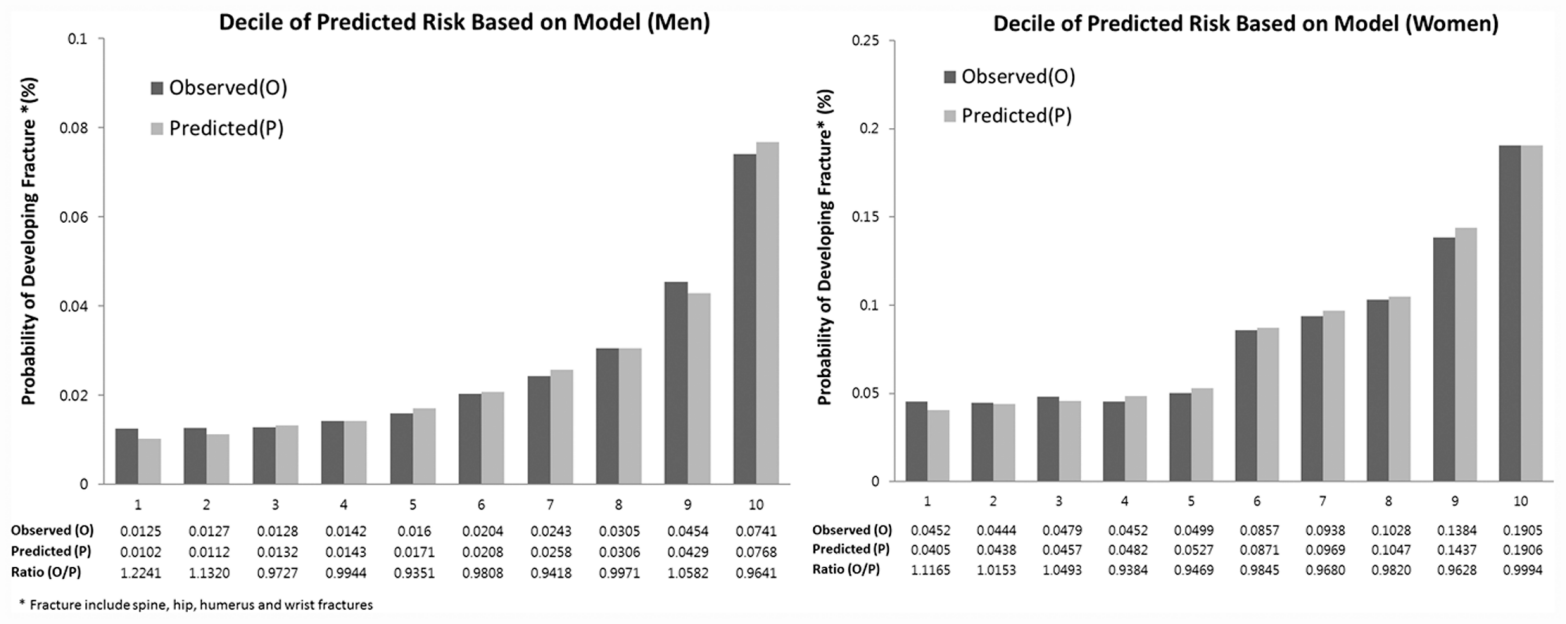
Predicted and observed risk for osteoporotic fracture at 7 years by 10^th^ of predicted risk using KFRS in the validation cohort.

The mean predicted scores after applying the KFRS with the observed risks at 7 years within each 10th of predicted risk were compared to assess calibration of the models in the validation sample (Fig 2). Close correspondence was observed between predicted and observed 7 year risks within each model 10^th^ for overall fracture. For example, in the top 10th of risk in women, the mean predicted 7-year risk of fracture was 19.06%, and the observed risk was 19.05%. The ratio of predicted risk to observed risk in this 10^th^ was 0.999, indicating almost perfect calibration (a ratio of 1 indicates perfect calibration, that is, no underestimation or overestimation). In men, the predicted event calculated from the KFRS in the top 10^th^ was slightly higher than the observed event (observed/predicted ratio: 0.964). The C statistics for the KFRS in the validation cohort were 0.68 for men and 0.65 for women, indicating that the discriminatory power of the KFRS is moderate.

## Discussion

Using data from a nationwide retrospective cohort of Korean men and women aged ≥ 50 years, we developed a novel predictive model called the KFRS to provide an easy method to estimate individual risk of osteoporotic fracture based on routinely available clinical information. More than 19,840 osteoporotic fractures occurred in the development cohort of more than 359,253 Koreans during the 7 years. The KFRS was developed based on these data. Our new model does not require laboratory testing or clinical measurements. All variables used in the model were collected from self-administered questionnaires that could be easily obtained from an individual at a primary care setting. The risk factors included age, BMI, history of recent fragility fracture, lack of regular exercise, higher alcohol intake, current smoking, recent use of oral glucocorticoid, history of rheumatoid arthritis, and use of medication or disease causing a low BMD. This model performed well when compared to the actual osteoporotic fracture cases in an independent sample cohort from which data had not been used to develop the algorithm. Previous studies related to osteoporotic fracture risk in Asian populations have been small community cohort studies [21, 22]. Our study may be the first nationwide study using an Asian cohort to develop a new risk prediction model.

Our new model can be used at a population level to identify high risk patients and support the clinical guidelines in Korea. The algorithm can be used for self-assessment at a web based calculator (http://www.nhis.or.kr) without BMD measurement. It can help inform patients regarding their absolute risk, so they can recognize the need for treatment. Current threshold interventional approaches based on BMD in Korea may result in under-treatment of high risk osteopenic patients. Further study is required to determine the threshold of individual absolute risk at which intervention will become cost-effective.

Most risk factors for fracture identified in the present study have also been identified in several recent meta-analyses of other population-specific cohorts [7, 8, 13, 31, 32]. Age contributes to fracture risk independently of BMD, and changes in age are approximately seven-fold more important than changes in BMD in another ethnic cohort [31]. In the present study, HRs were 5.66 and 4.72 for men and women aged 80–89 years, respectively, compared to those aged 50–59 years. Although only 2.3% of the cohort had a BMI < 18.5 kg/m^2^, it was identified as a significant independent risk factor for fracture. The mean BMI of the study participants was 23.9 kg/m^2^ in men and 24.3 kg/m^2^ in women, which was within the overweight BMI range for adult Asian women[24].

Recent fragility fracture was the strongest predictor of osteoporotic fracture after adjusting for age and BMI, confirming the results of earlier studies showing that a history of fracture at any site significantly increases the risk of future fracture[13, 33]. Kanis et al. have reported that osteoporotic fracture risk increases approximately two-fold in subjects with a previous fracture compared with those without a previous fracture in a meta-analysis[13]. No difference in the risk ratio was detected between men and women. In addition, other studies in postmenopausal women have found that the risk of subsequent vertebral fracture within 1 year of the original event is up to five times[34]. In our study population, the HR in men was higher than in other studies. In addition, a significant difference was detected between men and women (HR, 3.53 in men and 1.83 in women). Because we analyzed the history of fragility fracture within the past 2 years, HR for subsequent fracture could be higher than in other studies despite the low prevalence. However, the reason for the higher HR of recent fragility fracture in men remains unclear. As most studies have been conducted in women, further studies targeting men are needed.

Long-term use of corticosteroids is a known risk factor for fracture[12]. The range of RR for osteoporotic fracture is 2.63–1.71 in Western societies. In our study population, the hazard ratio was slightly lower (HR, 1.87 in men and 1.51 in women). High alcohol intake confers a significant risk of future fracture. In a Caucasian study, alcohol intake of more than 4 units per day increased the osteoporotic fracture risk (HR, 1.81 in men and 1.38 in women) [8]. In our study, high alcohol intake (≥ 5 units/day in men and ≥ 3 units/day in women) had a similar risk (HR, 1.37 in men and 1.2 in women). Current smoking is associated with significantly increased risk of osteoporotic fractures in both men and women, with risk being significantly higher in men than in women [10]. However, in our study, the risk was higher in women than in men (HR, 1.08 in men and 1.15 in women). Other contributing risk factors, such as lack of exercise[32], rheumatoid arthritis[31], and secondary osteoporosis[35] had slightly lower HRs compared to those of previous studies. Rheumatoid arthritis has been previously identified as a significant risk factor for any fracture[12], but it was significant only in men in this study. Ethnic group differences and other population-specific variables may be responsible for discrepancies between the results of our study and those of previous studies in other cohorts. Some variables known to be clinical risk factors for fracture in previous Korean and other Asian cohorts[21, 22] were not identified in the present study, including menopause, consumption of dairy products, history of one or more falls in 12 months, use of walking aids, and being housebound.

We did not include BMD as a result because it was not captured in the registry data. Our findings indicate that using clinical risk factors is sufficient to predict the risk of fracture. Adding BMD to the risk factor assessment would improve the osteoporotic fracture prediction. However, a recent review on the performance of osteoporosis absolute fracture risk assessment instruments by Nayak et al.[36] has reported that risk assessment instruments without BMD component have good calibration, similar to the proportion of risk assessment instruments with a BMD component. Therefore, the probability of fracture predicted from a risk factor assessment alone is sufficient enough to identify high risk patients who need treatment from subjects who have a combination of independent risk factors with high predictive value, such as history of fracture, advanced age, and low BMI. A risk factor assessment provides a far more amenable option than measuring BMD, particularly at primary care setting that often has limited access to dual-energy X-ray absorptiometry technology. However, clinical trials are needed to demonstrate that anti-osteoporosis drug treatment can prevent fractures in women selected for therapy based on these clinical risk factors alone.

A particular strength of our study is its nationwide cohort design based on an analysis of a large representative population from a validated database. Our main outcome was hip, vertebral, humeral, or distal radial fracture recorded by a clinician. Osteoporotic fracture incidence per 1000 person-years in our study was 3.61 in men and 12.09 in women. Our previous study using registry data including nationwide information compiled by the Korean government reported that the fracture incidence per 1000 subjects was 6.31 in men and 20.53 in women aged ≥ 50 years in 2012 (submitted data). Our rates of hip fracture were also lower than those reported in other studies using nationwide registry data[27]. This difference might be due to different operational definitions of fracture. In our study, we not only used ICD-10 codes but also used site-specific fracture reduction or fixation to identify osteoporotic fractures. Therefore, there might have been some over-estimates in previous studies because they only used ICD codes. The lower fracture incidence in our study may have resulted from characteristics of our cohort. Our study was aimed at subjects who had regular health screening and who completed a self-questionnaire. Therefore, we might have excluded subjects admitted to a nursing facility or acute-care hospital and those who had serious disease causing immobilization, such as stroke, Parkinson’s disease, and cancer; i.e. high risk subgroup for osteoporotic fracture. Our subjects were relatively healthy and had paid more attention to their health.

Our study had several potential limitations. First, there might be selection bias, as the participants were healthier than non-participants, even though the population was randomly selected among the national health examination-based cohort. However, considering that a fracture risk assessment tool is usually used in a primary care setting, our population may represent the general population who visits the primary care clinic where the model is likely to be used. Second, we did not have information on all risk factors for fracture (such as BMD, history of fall, or diet), which would improve the accuracy of the prediction for an individual patient. Third, although a split-cohort development/validation procedure was used, the most trustworthy approach to demonstrate the usefulness of a predictive model is to apply the model to an independent population. This model has not been validated in an independent population. A potential limitation of our validation is that there might have been selection bias as in the development cohort. Therefore, an additional validation study in a population unrelated to the development cohort to compare our model with other fracture risk assessment tools, such as FRAX, is currently under way. Finally, as we did not confirm fractures by radiology, we could not detect asymptomatic vertebral fractures. Therefore, fracture risk might have been underestimated due to the restrictive definition of a clinical vertebral fracture.

In conclusion, we developed the KFRS as a novel predictive model to predict the risk of osteoporotic fracture over 7 years in Koreans. The three most important risk factors for osteoporotic fracture in Koreans were advanced age, history of recent fragility fracture, and recent use of oral glucocorticoids. Our model will allow optimal risk assessment to identify Korean patients who would obtain the greatest benefit from treatment. It will be particularly useful in primary-care settings where there is limited access to instruments for measuring BMD. Further studies are needed to test the performance of our model in other Korean populations and to determine the interventional threshold based on the fracture probability in the Korean population.
